# Hospitalizations and re-hospitalizations at the end-of-life among cancer patients; a retrospective register data study

**DOI:** 10.1186/s12904-024-01370-1

**Published:** 2024-02-13

**Authors:** J. Singh, E. K. Grov, M. Turzer, A. Stensvold

**Affiliations:** 1 Department of Oncology, Østfold Hospital Trust, PO Box 300, Graalum, 1714 Norway; 2 Faculty of Health Sciences, Oslo Metropolitan University, St.Olavs Plass, PO Box 4, Oslo, 0130 Norway

**Keywords:** Hospitalizations, Re-hospitalizations, Palliative care, End-of-life care, Quality indicators

## Abstract

**Background:**

Patients with incurable cancer are frequently hospitalized within their last 30 days of life (DOL) due to numerous symptoms and concerns. These hospitalizations can be burdensome for the patient and the caregivers and are therefore considered a quality indicator of end-of-life care. This retrospective cohort study aims to investigate the rates and potential predictors of hospitalizations and re-hospitalizations within the last 30 DOL.

**Methods:**

This register data study included 383 patients with non-curable cancer who died in the pre-covid period between July 2018 and December 2019. Descriptive statistics with Chi-squared tests for the categorical data and logistic regression analysis were used to identify factors associated with hospitalization within the last 30 DOL.

**Results:**

A total of 272 (71%) had hospitalizations within the last 30 days of life and 93 (24%) had > 1 hospitalizations. Hospitalization was associated with shorter time from palliative care unit (PCU) referral to death, male gender, age < 80 years and systemic anticancer therapy (SACT) within the last 30 DOL. The most common treatment approaches initiated during re-hospitalizations remained treatment for suspected or confirmed infection (45%), pleural or abdominal paracentesis (20%) and erythrocytes transfusion (18%).

**Conclusion:**

Hospitalization and re-hospitalization within the last 30 DOL were associated with male gender, age below 80, systemic anticancer therapy and suspected or confirmed infection.

## Introduction

Several health service quality indicators have previously been identified as important to quality of care at the end-of-life (EOL) for cancer patients [1]. These indicators include the following five: frequency and duration of hospital admissions, intensive care unit (ICU) utilization, deaths in hospital and palliative care unit (PCU) utilization [1, 2]. Measuring these quality indicators can provide insights into areas where the quality and timing of health care provided is not optimized, and subsequently enabling continual improvement of quality of care and priorities to be set [3, 4]. Cancer patients are frequently hospitalized within the last 30 days of life (DOL) [5, 6] and the causes for admissions are often related to the cancer diagnosis or treatments, such as pain, infection or dyspnea [7, 8]. To our knowledge, factors associated with repeated hospitalizations within the last 30 DOL have to a lesser degree been explored. In this study, we hypothesized that certain disease-specific symptomology, demographic and logistic factors are increasing the risk of acute hospitalizations and re-hospitalizations within the last 30 DOL. Identifying potential risk factors for both hospitalizations and re-hospitalizations could contribute to improved health service quality by reducing the rate of burdensome acute re-hospitalizations and futile costly treatment close to death. Therefore, our aims were twofold; first, to assess the five health service quality indicators previously identified as important to quality of care at the EOL. Secondly, to identify risk factors associated with hospital hospitalizations and re-hospitalizations within the last 30 DOL, and describe the inpatient health care utilization during these hospitalizations. Additionally, differences in survival in relation to hospitalizations were assessed.

## Methods

### Material

We conducted a retrospective review of medical records for all patients who died in the pre-covid period between July 1, 2018, and December 31, 2019 and who were treated at the Oncology Department at a hospital in the southern part of Norway in the same period. Patients were eligible for the study if the malignant disease was documented in the patient record to be incurable and non-hematological. Since pediatric patients and patients with primary gynecologic cancer, head and neck cancer, pulmonary and neuro malignancies were treated in other departments, we did not include those. All hospitalizations within or extending into the last 30 DOL were mapped. Emergency department (ED) visits without subsequently hospitalizations were not included because they are mainly observational stays. Information on main cause of referral and main diagnosis during hospitalization was determined and classified by the first author by reviewing the medical records, and subsequently confirmed by co-author AS. Procedures of less intrusive character and related medications, such as fluid therapy and pain and nausea management, were not registered. These medications are frequently administered and altered in these patients, both in a community-based health care setting and during hospitalizations, independently of main cause of admission.

### Statistical considerations

Dichotomization was performed based on the presence or absence of hospitalization within the last 30 DOL and served as dependent variable. For categorical variables, Pearson`s chi-squared test was used for group comparisons. Cox regression analysis and Kaplan–Meier test were used for exploring significance and differences in survival time. To analyze which factors were significantly associated with hospitalization at EOL, we used logistic regression analysis. All patients had a cancer diagnosis; however, particular cancer type was excluded from the regressions analysis due to low frequency in many of the cancer types. Significance level was defined as < 0.05 and all testes were two-tailed.

## Results

**Fig. 1:**
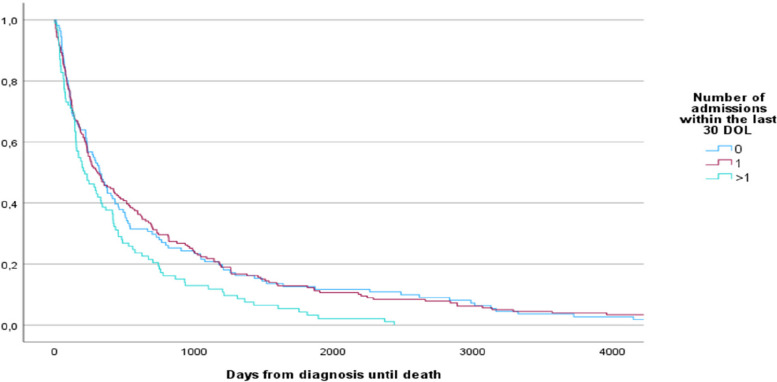
Kaplan–Meier graph of time from diagnosis until death among those never hospitalized, hospitalized once and those hospitalized more than once during the last 30 DOL

In total 416 patients were identified from record search and among these 383 patients were analyzed. The reasons for exclusion were: malignant entities treated at other departments or other hospitals (*n *= 19), curative intention of treatment (*n* = 11) and hematological malignancies (*n* = 3). The majority of patients in our study had gastrointestinal cancer (*n* = 200) and were males (*n* = 237). A total of 272 (71%) had hospitalizations within the last 30 days of life and 93 (34%) of these had > 1 admissions (range 2–4). Mean time spent in hospital within the last 30 DOL was 8 days (range 0–29), which makes up 27% of the time. Mean duration of each stay was 5.6 days (*n* = 386). Median interval from first hospitalization within the last 30 DOL to re-hospitalizations was 7 days. Time from last hospitalization and last out-patient appointment to the first hospitalization within the last 30 DOL, was 41 days (median) and 29 days (median) respectively. Patients with two or more hospitalizations (*n* = 93) within the last 30 DOL had a mean age of 68 compared to 72 among those only hospitalized once (*p* = 0.014). Interval from diagnosis until death was significantly shorter among those with two or more hospitalizations, compared to those with only one hospitalization (mean 443 vs 799 days, *p* = 0.008, Fig. 1). Demographic and clinical characteristics of patients hospitalized and those not hospitalized within the last 30 DOL is found in Table 1.

**Table 1:** Characteristics of patients hospitalized and those not hospitalized within the 30 last days of life

Demographic and clinical variables	Hospitalization within the last 30 days of life		
	Yes	No	
	*n* = 272 (%)	*n* = 111 (%)	*p*
Gender			
Male	178 (65%)	59 (53%)	0.025
Female	94 (35%)	52 (47%)	
Age at death (years)			
Mean	70	73	0.150
Median	72	74	
Patients with minor children			
Yes	21 (8%)	8 (7%)	0.863
No	251 (92%)	103 (93%)	
Primary malignancy			
Colorectal	52	30	
Pancreatic	42	13	
Prostate	40	14	
Breast	24	17	
Urothelial	18	5	
Bile duct	17	5	
Kidney	15	9	
Malignant melanoma	15	3	
Esophageal	14	2	
Ventricular	11	6	
Hepatic	8	0	
Cancer of unknown primary cite	6	5	
Others	10^g^	2^h^	
Multiple primary malignancies			
Yes	13 (5%)	10 (9%)	0.114
No	259 (95%)	101 (90%)	
Anticancer treatment lines^i^			
Never treated	67 (25%)	41 (37%)	0.015
Treated	205 (75%)	70 (63%)	
One line	79	38	
Two lines	62	10	
Three lines	24	8	
Four lines or greater	40	14	
Anticancer treatment within the last 30 DOL^j^			
No	168 (62%)	86 (77%)	0.003
Yes	104 (38%)	25 (23%)	
Chemotherapy	30^ab^	2	
Targeted therapy	22^acd^	2^f^	
Check-point-inhibitors	3	1	
Endocrine therapy	46^bce^	21	
Radiation therapy	18^cde^	0	
Palliative care unit referral			
Yes	184 (68%)	79 (71%)	0.500
No	88 (32%)	32 (29%)	
Interval from PCU referral until death			
Mean (days)	99	181	< 0.001
Median (days)	57	102	
Use of PCU within the last 30 DOL^j^			
Yes	155 (57%)	46 (41%)	0.006
No	117 (43%)	65 (59%)	
Interval from diagnosis until death			
Mean (days)	677	763	0.471
Median (days)	275	326	

^a^Three patients received both chemotherapy and targeted therapy

^b^Two patients received both chemotherapy and endocrine therapy

^c^Five patients received both targeted therapy and endocrine therapy

^d^One patient received both targeted therapy and radiation

^e^Four patients received both endocrine therapy and radiation

^f^One patient received both targeted therapy and endocrine therapy

^g^Seven patients with NEC (neuroendocrine carcinoma), two with NET (neuroendocrine tumor) and one with duodenal cancer

^h^One patient with NEC and one with sarcoma

^i^Endocrine therapy included as treatment line

^j^Days of life

Patients with 2 or more hospitalizations (*n* = 93) were more likely to be living at a nursing home facility by hospitalization number two, three and four (*n* = 114) compared to index hospitalization and those with only one hospitalization (27% vs 13%, *p* < 0.001). Utilization of nursing home care services was not significantly higher prior to re-hospitalization in those still living at home compared to the index hospitalization and those with only one hospitalization (72% vs 52%, *p* = 0.100). Out-of-hours municipal medical center was involved in 11% of the index hospitalizations and those with only one hospitalization within the last 30 DOL (*n* = 272), decreasing to 4% in the 114 re-hospitalizations.

Suspected or confirmed infection (30%), bronchopulmonary insufficiency (13%), pain (12%) and bile obstruction (8%) remained the most common main diagnosis in re-hospitalizations (*n* = 114). In hospitalization number three or four (*n* = 21), suspected or confirmed infection was the main diagnosis in half of the hospitalizations. Characterizations of referrals and hospital stays within the last 30 DOL is found in Table 2.

**Table 2:** Characterizations of referrals and hospital stays within the last 30 days of life

Demographic and clinical variables	Hospitalizations	
	*n* = 386	%
Care setting prior to hospitalization		
No home care nursing	139	36%
Home care nursing < 1/day	36	9%
Home care nursing > 1 /day	149	39%
Nursing home residency	62	16%
Origin of admission		
Direct admission	149	39%
Nursing home	53	14%
PCU	43	11%
Local hospital (not in connection with patient appointment)	42	11%
Family doctor	39	10%
Out-of-hours municipal medical center	34	9%
Out-patient clinic appointment	22	6%
Other hospital	4	1%
Admission in out-of-office hours		
Yes	166	43%
No	220	57%
Planned admission		
Yes	35	9%
No	351	91%
Main complaint/cause of referral		
Fever/infection	69	18%
Dyspnea	67	17%
Pain	64	17%
Neurological	48	12%
General decreased condition	32	8%
Nausea/vomiting	21	5%
Ascites/edema	20	5%
Jaundice	14	4%
Bleeding	15	4%
Invasive catheter/tube problem	11	3%
Diarrhea	6	2%
Obstipation	4	1%
Other^a^	15	4%
Main diagnosis/cause of hospitalization		
Infection/fever	106	27%
Bronchopulmonary insufficiency	41	11%
Gastrointestinal passage disturbance	39	10%
Pain management	37	10%
Neuropathy or plexopathy	26	7%
Bile obstruction	23	6%
Nutritional or metabolic disorder	20	5%
Bleeding	19	5%
Renal insuffiency/hydronephrosis	14	4%
Ascites	13	3%
Catheter or tube problem	10	3%
Coronary disease/heart failure	8	2%
Ischemic stroke or TIA	8	2%
Venous thromboembolism	6	2%
Other^b^	16	4%
Destination when discharged		
Nursing home facility	162	42%
Home	122	32%
Death during hospitalization	102	26%

^a^Includes two patients admitted for planned surgery, three with kidney failure (incidental findings), one patient admitted for planned in-patient systematic anticancer treatment administration, one patient with cardiac arrest, one with anuria, one with suspected allergic reaction to chemotherapy administration, three with dysphagia, one with urinary retention, one with anxiety and one for a second opinion cancer assessment

^b^Includes four patients with liver failure, two with disseminated intravascular coagulation (DIC), two with cardiac arrest, two with allergic reaction, one with opioid overdose, one with planned surgical tumor debulking, one with hepatic encephalopathy, one with irritation of the diaphragm due to liver metastasis, one with cardiac rhythm disturbance and one with urinary retention

Antibiotics (45%), pleural or abdominal paracentesis (20%) and erythrocytes transfusion (18%) remained the most common treatment approaches initiated during re-hospitalizations (*n* = 114) compared to index hospitalization and those with only one hospitalization (*n* = 272). There was a decrease in imaging utilization in re-hospitalizations compared to index hospitalization and in those with only one hospitalization; Magnetic Resonance Imagination (MRI) (4% vs 10%), Ultrasound (31% vs 39%) and Computer tomography (32% vs 51%). Details on imaging utilization and new treatment approaches initiated in 386 hospitalizations within the last 30 DOL is found in Table 3. Regression analysis on factors associated with hospitalization within the last 30 DOL is presented in Table 4. Age below 80, male gender and SACT the last 30 DOL remained associated with hospitalizations in the analysis.

**Table 3:** Imaging utilization and new treatment approaches initiated in those hospitalized only once and those with two or more hospitalizations within the last 30 days of life

Procedures	Hospitalizations within the last 30 DOL	
	Once (*n* = 179)	≥ 2 (*n* = 93)^a^
	Number of procedures	Number of procedures
Imaging utilization		
X – ray	110	47
Computed Tomography^d^	90	49
Ultrasound	70	36
Magnetic resonance imaging	11	15
Other	2^e^	0
Treatment approaches		
Antibiotics	98	88
Erythrocyte transfusion	49	46
Pleural paracentesis	18	20
Abdominal paracentesis	25	16
Invasive nutrition support	13	4
Radiation therapy	3	13
Gastroscopy	10	5
ERCP/PTC^b^	4	10
Platelets transfusion	9	1
MICU^c^	7	3
Biopsy	3	3
Nephrostomy	3	5
Non-invasive ventilation	4	4
Spinal/epidural punction	3	3
ICU	3	2
Abscess drainage	1	3
Colo-/rectoscopy	2	1
Bronchoscopy	1	2
Major surgery	2	1
Invasive mechanical ventilation	1	2
Plasma products	2	0
Terminal sedation	1	0
Other	1^f^	2^g^

^a^Index hospitalization included

^b^Endoscopic retrograde cholangiopancreatography/Percutaneous transhepatic cholangiography

^c^Medical intensive care unit

^d^Positron Emission Tomography – Computed tomography (PET/CT) and Computed tomography simulation for radiation therapy included

^e^Three interventional angiographies

^f^One dialysis

^g^One cystoscopy in anesthesia and one nuclear glomerular filtration rate (GFR) test

**Table 4:** Regression analysis for the impact of demographic and clinical variables on hospitalizations within the last 30 DOL

Covariates	Standard error	*p*-value*	HR	95% CI^a^	
				Lower	Upper
Age at death					
< 65	.323	.019	2.137	1.135	4.023
65–79	.277	.027	1.845	1.072	3.175
≥ 80^b^					
Gender					
Male	.237	.020	1.735	1.089	2.763
Female^b^					
SACT^c^ during last 30 DOL^d^					
Yes	.488	.001	4.814	1.850	12.526
No^b^					

^a^95% CI for Hazard ratio

^b^Reference group

^c^Systemic anticancer therapy

^d^Days of life

^*^Uncorrected

## Discussion

This study assesses the health care utilization in the last 30 DOL in an unselected cohort in a cancer department setting. Our aims were twofold; first, we examined five health service quality indicators previously identified as important to quality care at the EOL. Secondly, we identified the factors leading to hospitalizations and re-hospitalizations within the last 30 DOL and described the inpatient health care utilization during these hospitalizations.

A total of 383 patients were included and among these 272 (71%) were hospitalized with a total of 386 admissions within the last 30 DOL. This rate is higher than findings in several other countries where frequencies vary from 43.2 to 62.6% [9, 10]. Only SACT within the last 30 DOL, age below 80 and male gender remained significant predictors for hospitalizations in the regression analysis. The association between SACT and hospitalizations have previously been well established [11–13]. Our findings are in accordance with this association as the vast majority of patients receiving chemotherapy or targeted therapy within the last 30 DOL also where hospitalized.

The proportion of patients with more than one hospitalization within the last 30 DOL was considerable higher at 24% in this study, compared to findings in several other European countries where frequencies vary from < 1% and 11.7% [3, 11]. The reason for our higher rate is not easy to explain. It may be attributed to the characteristics of the patient cohort or local treatment tradition. Our findings are also higher than suggested performance standard stating that less than 4% should have more than one admission within the last 30 DOL [14].

Median total bed days of eight within the last 30 DOL is comparable to findings of seven and nine days in other studies [5, 15]. Hospital death rate of 38% is also similar to findings in other European countries where frequencies vary from 29.4 to 67% [5, 9, 16]. Referral to PCU was not associated with fewer hospitalizations within the last 30 DOL in this cohort, contrary to findings in several other studies [17–19]. However, time from referral to PCU until death was significantly shorter in those hospitalized. With an interval of only 56.5 days (median) from referral to PCU until death, there is a limited opportunity window for interventions and facilitation of the last part of life. Early palliative care (i.e. > 6 months prior to death) decreases the risk of receiving hospital care and dying in hospital and increases the probability to receive supportive home care nursing in the last month of life [18]. Taking into account that those not hospitalized in our study were referred only 3 months (median 102 days) prior to death, there seems to be potential for improvement in timing of PCU utilization. Our analysis also showed that whether the patients were referred to PCU prior to the last 30 DOL or not, had no impact on the frequencies of procedures initiated during those last 30 DOL. This also emphasizes the importance of adequate timing of referral to PCU. Our findings of ICU utilization at 4% can be considered in the lower range compared to findings in other Western countries where rates varied between 3.5% and 27.1% [9]. This variation might be attributable to differences in ICU capacity, but also cultural differences in PCU utilization between countries. Previously suggested performance standard of no higher than 4% ICU utilization within the last 30 DOL [14], complies with our findings.

A significant share of the referrals happened out-of-office-hours (43%), similar to findings in other studies [20, 21]. These acute referrals are patient- or caregiver initiated or often involve physicians not known to the patient. Unacquaintance with the patient, the medical history and the field of palliative care, can lower the barrier for referral and hospitalizations [22]. The involvement of General Practitioners (GPs) in connection with hospitalizations at the end-of-life seemed to be quite low and decreased with increasing numbers of re-hospitalizations. Although most patients were living at home when admitted, there was an increasing share of patients living in nursing homes upon re-hospitalizations, which can explain the descending involvement of GPs in re-hospitalizations. Previous studies have shown that palliative care provided by GPs is associated with less time spent in hospital, fewer hospitalizations and non-institutional deaths [3, 23, 24].

Reasons for referrals and main diagnosis during hospitalizations were similar to findings in other studies on cancer patients [7, 20, 21]. Most common treatment approaches initiated during re-hospitalizations within the last 30 DOL were treatment for suspected or confirmed infection, drainage or erythrocyte transfusions. This highlights the importance of advance directives (AD) and EOL care planning. The need of drainage and erythrocytes transfusions can to a certain degree be predicted and planned at the later stage of disease, resulting in minimalized discomfort and time spent in hospital. Also, fever or infection as the most frequent cause of re-hospitalizations in our cohort, emphasizes the challenges in potentially time-critical decision-making processes in pre-hospital settings.

We do not know the clinical implications of the high imaging utilization in this cohort, mainly due to the retrospective character of the study with data from diseased patients with no description on the intention of imaging. As far as we know, such study has not been performed in other comparative hospitals and we therefore have no reference on the use of imaging in this target group. Advanced imaging, such as MRI, might lead to appropriate palliative treatment approaches and de-escalate the intensity of care by reducing futile treatment. However, it can also distract patients from achievable end-of-life goals and are time-consuming and costly procedures [25].

Our findings should be interpreted in the context of its limitations. Although comprehensive data was gathered, some relevant data was not available due to the retrospective design of the study. Access to informal care, patients- and caregivers’ preferences and activities of daily life are factors, which can influence end-of-life management and utilization of health care [13, 22]. A prospective design including qualitative data and patient-reported outcomes might give a more precis description of factors leading to hospitalizations and differences in treatment intensity. We did neither map preexisting comorbidities, cancer stage and details regarding histology of cancers and mutations with treatment and prognostic implications. These factors can also influence the decision-making process regarding treatment intensity and health care utilization [13]. Particular cancer types were too low in numbers to be analyzed as risk factors. However, the association between health care utilization and different cancer types has previously been explored [8, 19, 26]. Cancer patients at the end-of-life often have multiple complaints. By identifying solely main symptom and diagnosis, we probably do not encompass the complex clinical picture and the need of treatment approaches in these patients. The high number of procedures initiated unrelated to main diagnosis during hospitalization in our study, such as abdominal and pleural paracentesis, illustrates the symptom complexity in these patients. However, by conducting a journal review we were able to uncover reasons for hospitalizations more precisely, compared to classification by database coding solely, which can lead to misclassification in a multifaceted hospitalization trajectory. We did not map rate of advanced directives and limitations on life-sustaining treatments, which are associated with improved quality of care at the end-of-life [27–29]. These factors have most likely influenced re-hospitalization rates and treatment procedures initiated in this cohort. Actual cause of death was not registered, but in this population, the cancer diagnosis is likely to contribute to death. Since our study includes hospitalizations extending into the last 30 DOL, days hospitalized preceding the last 30 DOL have been included. This makes direct comparison of days spent in hospital somewhat inaccurate. Patients with suspected or confirmed incurable cancer diagnosis never assessed at the Cancer Department, were not included in our study. Many of those may have been regarded as not eligible for SACT and therefore not referred to an oncologist from their GPs or nursing home doctors. This may represent a potential selection bias to our cohort, as we do not know the frequency of hospitalizations at EOL in those patients. On the other hand, real-world evidence studies, including patient registries and electronic health record studies, can provide valuable information on treatment practices and patient characteristics. Ultimately, this information can be used in guidance on treatment decisions and increase end-of-life quality. Further, comparing our immediate pre-pandemic findings to post-pandemic, could illuminate the consequences of the pandemic on health care services utilization in this vulnerable group, including hospitalizations, anti-cancer therapy and PCU-utilization.

## Conclusion

Hospitalizations rates within the last 30 DOL were high, and timing of PCU-utilization not optimized. Male gender, age below 80 and systemic anticancer therapy were associated with hospitalizations. Antibiotics, pleural or abdominal drainage and transfusions were the most common treatment approaches in re-hospitalizations, emphasizing the importance of advance care planning.

## Data Availability

The data that support the findings of this study are available from the corresponding authors, upon reasonable request.
